# Enhanced lipid metabolism reprogramming in CHF rats through IL-6-mediated cardiac glial cell modulation by digilanid C and electroacupuncture stimulation combination

**DOI:** 10.3389/fcell.2024.1424395

**Published:** 2024-09-03

**Authors:** Yun Liu, Xiao Sun, Mingqian Yuan, Zhi Yu, Qun Hou, Zhengxu Jia, Tiancheng Xu, Bin Xu

**Affiliations:** Key Laboratory of Acupuncture and Medicine Research of Ministry of Education, Nanjing University of Chinese Medicine, Nanjing, China

**Keywords:** digilanid C-electroacupuncture stimulation, chronic heart failure, cardiac glial cells, lipid, glutamatergic

## Abstract

**Background:**

Cardiac lipid metabolism reprogramming is recognized as a critical pathological factor in the progression of chronic heart failure (CHF). The therapeutic potential of digilanid C and electroacupuncture stimulation (ES) in enhancing lipid metabolism and cardiac function has been established. However, the optimal synergistic regulatory strategies of these interventions on cardiac lipid metabolism have yet to be elucidated.

**Methods:**

This study aimed to comprehensively evaluate the impact of a digilanid C-ES combination on cardiac steatosis remodeling in CHF. Assessments were conducted across various dimensions, including myocardial oxygen consumption, mitochondrial function, and lipid metabolism. Additionally, we sought to uncover the underlying neuromolecular mechanisms.

**Results:**

Our findings, at both molecular and morphological levels, indicated that the synergistic application of digilanid C and ES significantly inhibited myocardial fibrosis and steatosis. This combination therapy facilitated the repair of cardiac neuro-vascular uncoupling and induced a reprogramming of lipid metabolism. Notably, the digilanid C-ES combination ameliorated cardiomyocyte apoptosis and enhanced mitochondrial biogenesis in CHF, leading to a restructured energy supply pattern. Cardiac immunofluorescence analyses revealed the aggregation of cardiac glial cells (CGCs) at sites of abnormal neurovascular coupling, a response to cardiac lipid degeneration. This was accompanied by a marked reduction in the abnormally elevated expression of interleukin 6 (IL-6) and glutamatergic signaling, which correlated with the severity of cardiac steatosis and the aberrant activation of CGCs. The combined therapy was found to activate the Janus kinase 1 (JAK1)/signal transducer and activator of transcription 3 (STAT3) pathway, effectively attenuated lipid accumulation and over-recruitment of CGCs and deprivation of glutamatergic nerves.

**Conclusion:**

These findings underscore the potential of digilanid C and ES combination therapy as a novel approach to modulate the complex interplay between neurovascular dynamics and metabolic dysregulation in CHF.

## 1 Introduction

Lipids, as second messengers, are integral to signal transmission in heart cells and serve as vital bioenergy substrates for sustaining cardiac function (Borlaug et al., 2023). Metabolic alterations have been recognized as pivotal pathophysiological drivers of chronic heart failure (CHF), as evidenced by both preclinical models and clinical studies (Schiattarella et al., 2019; Tong et al., 2019). The reprogramming of lipid metabolism in response to cardiac dysfunction can exacerbate myocardial fibrosis and further decrease ejection fraction, highlighting the importance of cardiac energy rebalancing and the mitigation of myocardial damage through the dynamic monitoring and prompt treatment of lipid metabolism disorders (Lin et al., 2024).

The enlargement of abdominal subcutaneous adipocytes is associated with an increased risk of cardiometabolic disorders (Lavie et al., 2017). Visceral adipose tissue (VAT), including epicardial fat, can predict lipid metabolism anomalies in CHF patients, despite being more susceptible to altered metabolic profiles than upper body subcutaneous adipose tissue (iWAT) (Iacobellis, 2022). Epicardial adipose tissue can mediate detrimental effects on myocardial structure, function, and metabolism, and when combined with systemic inflammation, can lead to abnormal neurohormonal activation, autonomic dysregulation, and altered hemodynamic load (van Woerden et al., 2022). The complex signaling interactions between cardiomyocytes and epicardial fat, while complicating the pathophysiology of CHF, also offer new perspectives for therapeutic interventions.

The sodium-potassium pump (NKA) is a direct target of digilanid C, and its state change is critical for sustaining life’s metabolic processes (Kanai et al., 2021). The cardiac NKA-modulating effect of digilanid C is energy-dependent and capable of reorganizing energy patterns. In the context of cardiac insufficiency, digilanid C has been shown to reduce cholesterol and beta-lipoprotein levels while regulating coenzyme and adenosine nucleotide levels (Gorchakova et al., 1977). High doses of digilanid C have been associated with the accelerated development of foam cells, increased oxidized low-density lipoprotein (oxLDL) absorption, and atherosclerosis, suggesting its potential to control energy-transmitting molecules such as lipids and adenylates (Shi et al., 2016). Our pilot trial results indicate that electroacupuncture stimulation (ES) can enhance the targeted accumulation of digilanid C in the heart, thereby augmenting its efficacy (Liu et al., 2023). Additionally, ES has been shown to positively regulate systemic lipid metabolism, suggesting that a combination of digilanid C and ES may be particularly effective in reducing cardiac lipotoxicity and enhancing cardiac function.

Cardiac glial cells (CGCs), under disease stress, establish direct synapse-like connections through the cardiac intrinsic nervous system, which are involved in mediating the inflammatory response and ecological niche remodeling associated with myocardial stress (Kanemaru et al., 2023). CGCs, which can be identified by markers such as glial fibrillary acidic protein (GFAP), glutamate-aspartate transporters (GLAST), and glutamine synthetase (GS) (Kikel-Coury et al., 2021), are conserved across species (Scott-Solomon et al., 2021). This conservation implies that CHF therapeutic strategies targeting cardiac-connected CGCs hold significant clinical translational potential. Astrocytes, which share characteristics with CGCs, have been shown to influence lipid transport and metabolism, affecting an individual’s susceptibility to diet-induced obesity (Chen et al., 2023; Varela et al., 2021). Given these similarities, it is plausible that CGCs may control gene polymorphisms related to lipid metabolism and nutritional sensitivity to metabolic syndrome.

Our study demonstrates that aberrant increases in the number and activity of CGCs coexist with myocardial lipid accumulation and altered cardiac metabolism in CHF. The Janus kinase 1 (JAK1)/signal transducer and activator of transcription 3 (STAT3) pathway, mediated by interleukin 6 (IL-6), is implicated in the control of lipid accumulation in cardiomyocytes by CGCs. Overexpression of IL-6 leads to increased activity of the JAK1-related signaling pathway. The combination of digilanid C and ES mitigates the overstress of CGCs on cardiac lipotoxicity, improves the abnormal interaction of CGCs with cardiac glutamatergic nerves, and ultimately reduces lipid metabolic stress, leading to improved cardiac function. These findings reveal that the downregulation of the IL-6-driven JAK1/STAT3 axis by a combination of digilanid C and ES is a key mechanism for alleviating the pathogenesis of cardiomyocyte dyslipidemia, linking aberrant lipid metabolism in CHF to CGCs overactivation.

## 2 Materials and methods

### 2.1 CHF model rats

Two hundred Sprague Dawley (SD) rats, each weighed 200 g, sourced from the Model Animal Research Center of Nanjing University of Chinese Medicine (No. 1100112011052760, under grant SCXK(JING)2016–0006). Following a week of acclimatization, the rats were anesthetized with 3% isoflurane and intubated to maintain respiration. Chest expansion was facilitated using a chest opener to fully expose the heart. The left anterior descending branch of the coronary artery (LAD) was ligated with a 6–0 non-absorbable suture. Successful infarction was confirmed by an elevated or inverted T wave on the ECG and an elevated ST-segment potential of 0.1 mV (Tian et al., 2022; Tian et al., 2023b). The chest was then closed and sutured in layers. After 6 weeks of regular feeding, left ventricular ejection fraction (LVEF), and blood brain natriuretic peptide (BNP) levels were measured to validate the model’s efficacy. Following successful modeling, rats were randomly assigned to the model group, digilanid C group, ES group, or digilanid C-ES combination (digilanid C + ES) group based on LVEF.

### 2.2 Western blot

Equal amounts of heart protein (8 μg) were loaded onto a precast gel (Genscript) and subjected to electrophoresis at 140 V. The PVDF membrane was blocked in a 5% bovine serum albumin solution at room temperature for 1 hour. Primary antibodies were diluted and incubated at 4°C for 16 h. Details of the primary antibodies are provided. Corresponding secondary antibodies were incubated at 37°C. HRP-linked antibodies (1:2000, Signalway) were used for both rabbit IgG and mouse IgG.

### 2.3 Measurement of myocardial oxygen consumption

Rats were maintained at a constant core body temperature using a thermostat. They were restrained in a rat immobilizer for at least 20 min to minimize stress. The probe of an automated rat tail pressure measurement system (CSWY-3, Chengdu Instrument Factory) was fixed at the tail artery to ensure the rat’s tail was at rest during measurement. Measurements were averaged over five readings. Myocardial oxygen consumption was calculated as heart rate multiplied by systolic blood pressure (Fysekidis et al., 2019).

### 2.4 Oil red O staining

Tissue sections were rinsed with phosphate-buffered saline to reduce interference. A 60% isopropanol solution was prepared by diluting 100% isopropanol with deionized water. Slides were immersed in this solution for 2 minutes to enhance tissue permeability and ensure optimal staining. After drying, Oil Red O stain (Beyotime, China) was applied to the slides. The stain was protected from light exposure for 10 minutes before being washed off with a 60% isopropanol solution. The cell nucleus was then counterstained with hematoxylin for 1 minute, followed by a 10-min tap water rinse. Slides were mounted using glycerin gelatin (Beyotime, China) (Torous and Ly, 2022).

### 2.5 Immunofluorescence

Heart tissue was fixed in 4% paraformaldehyde for 24 h and then dehydrated in a 30% sucrose solution overnight. Primary antibodies against glial fibrillary acidic protein (GFAP) (1:200, Signalway, United States) and interleukin 6 (IL-6) (1:500, Signalway, United States) were applied and incubated at 4°C for 17 h. Excess primary antibodies were washed off with 0.1 M phosphate-buffered saline (Biosharp Life Sciences, China), followed by incubation with secondary antibodies (Alexa Fluor 488 goat anti-mouse and 594 goat anti-rabbit, 1:500, Abcam, United Kingdom) for 1 hour at 37°C. Images were captured using a fluorescence microscope (Olympus BX60, Japan).

### 2.6 Treatment intervention

Animals were anesthetized with 5% isoflurane for the duration of the EA procedure, maintaining anesthesia at 2% isoflurane. Digilanid C solution (Chengdu Bite Pharmaceutical Co., Ltd, 0.2 mg/mL, Batch No. H32021538) was administered via tail vein injection at a dosage of 0.1 mg/kg (Xue et al., 2017). Rats in the ES group received stimulation at left PC6 throughout the ES procedure. The digilanid C-ES combination group was treated with EA at left PC6 before tail vein injection with the same parameters as the ES group. Treatments were administered once daily, 5 days per week, with 2 days off, totaling ten sessions for each group (Han, 2023).

### 2.7 Data analysis

Data are presented as mean ± standard error. Paired t-tests were used to compare two groups after EA intervention, and independent t-tests were performed for comparisons between two groups. One-way analysis of variance (ANOVA) was used for multiple group comparisons. GraphPad Prism 8.0 (GraphPad Inc., United States) and SPSS 22.0 software (IBM Corp., United States) were utilized for all data analyses. A *p*-value of less than 0.05 was considered statistically significant.

## 3 Results

### 3.1 Synergistic effect of digilanid C and ES on myocardial oxygen consumption and cardiac function

**FIGURE 1:**
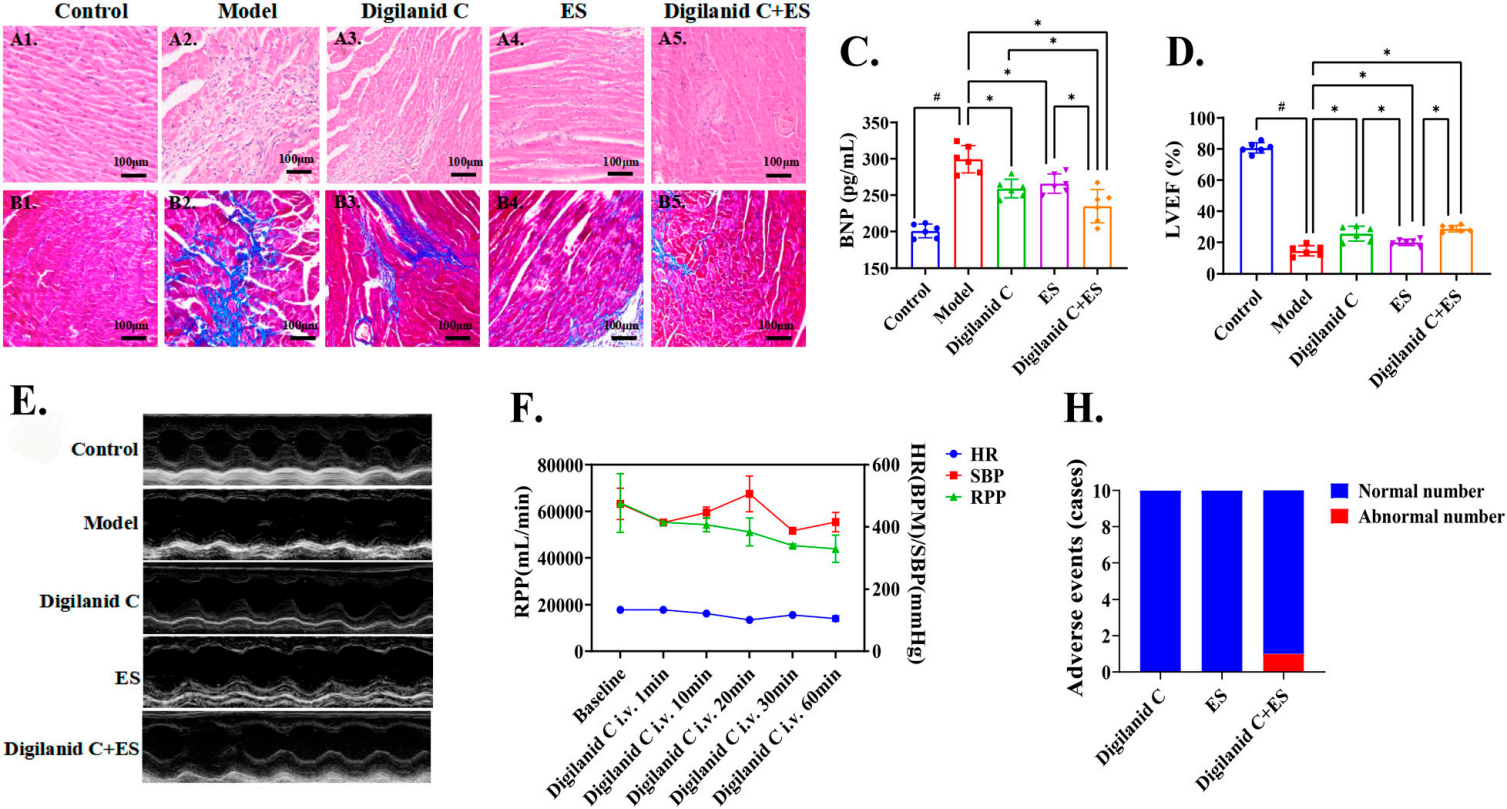
Digilanid C-ES combination effectively improves cardiac function and cardiac energy supply in rats with CHF. **(A)** Representative images of HE and **(B)** Masson staining of rat hearts in each group (×200 magnification). **(C)** The modulation advantage of digilanid C-ES combination on serum BNP in CHF rats. **(D)** Differences in LVEF and **(E)** representative images of cardiac ultrasound in LVEF in each group of rats. **(F)** Effect of sildenafil injection on myocardial oxygen consumption in rats after electroacupuncture intervention. **(H)** Adverse event statistics for each intervention group. ^#^ *p* < 0.05 vs. the normal control, ^*^ *p* < 0.05 vs. the model or another treatment group. ES, electracupuncture stimulation; LVEF, left ventricular injection fraction; BNP, brain natriuretic peptide; RPP, rate pressure product; HR, heart rate; SBP, systolic pressure.

Digilanid C or ES significantly ameliorated myocardial inflammatory accumulation and fibrotic degeneration, as evidenced by the histological assessments shown in Figures 1A1–4, 1B1–4. Notably, the combined treatment exerted the most pronounced effects, as illustrated in Figures 1A5, 1B5. The intervention groups demonstrated a dominant influence on ventricular remodeling and enhancement of LVEF, with comparable improvements in serum BNP levels, as depicted in Figures 1C–E. Concurrently, we observed that the combined intervention of digilanid C and ES substantially reduced myocardial oxygen consumption, thereby modulating the cardiac energy supply-demand balance, as shown in Figure 1F. Meanwhile, digilanid C combined with ES effectively improved the survival of CHF rats (Figure 1G). There has been only one case of premature ventricular within one and a half hours of intervention (our former experiment suggested that cardiac drug concentration peaks around 1 h) (Figure 1H).

### 3.2 Amelioration of cardiac lipotoxicity in CHF rats by the digilanid C and ES combination

**FIGURE 2:**
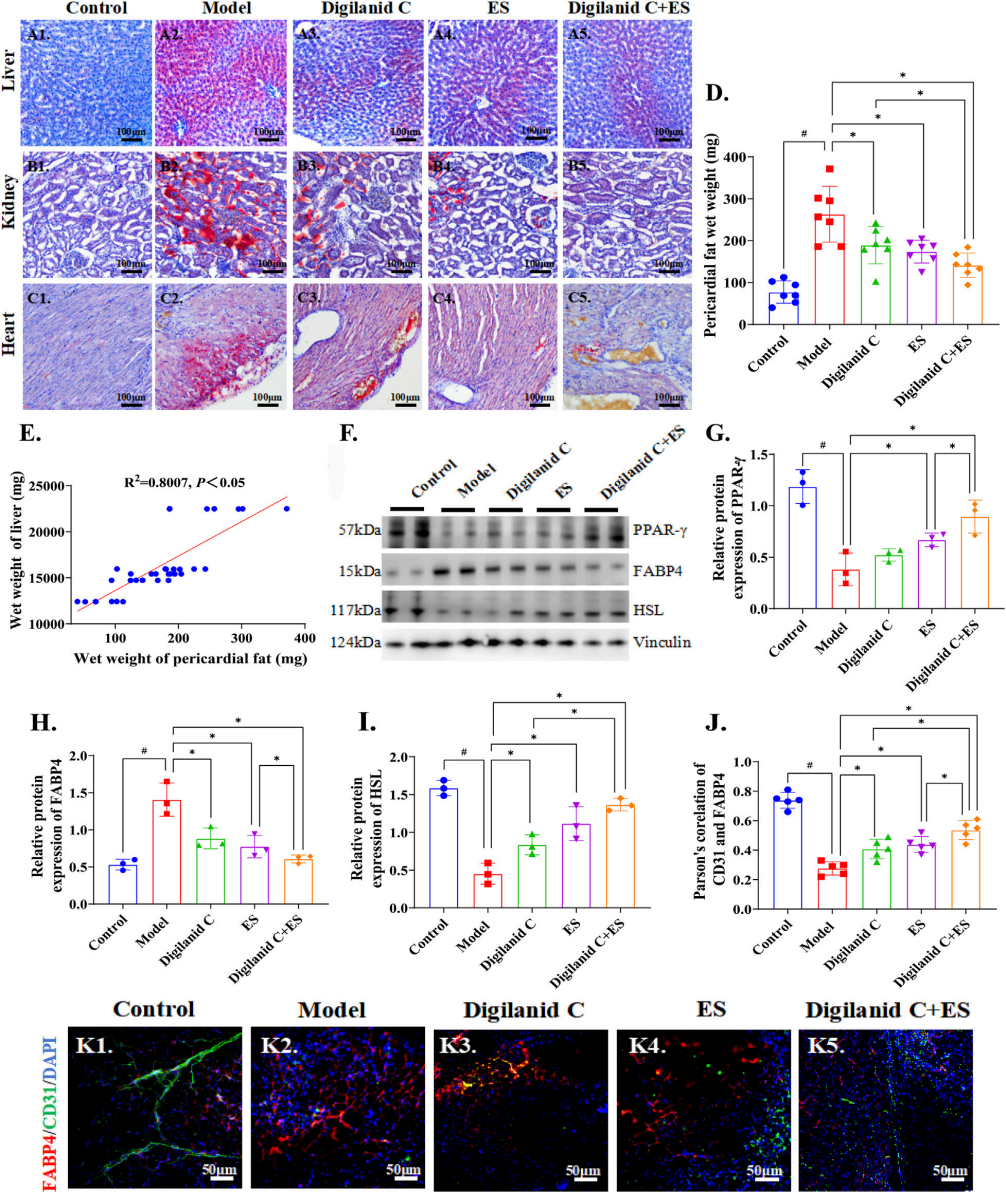
Digilanid C-ES combination to regulate cardiac fat overload. Typical representative plots of ORO staining of the liver in the **(A1)** normal, **(A2)** model, **(A3)** digilanid C, **(A4)** ES, and **(A5)** digilanid C-ES combination groups. Differences in the degree of fatty degeneration in kidney and **(C)** heart tissues in different groups. **(D)** Modulation of pericardial fat wet weight by different intervention groups. **(E)** Correlation analysis between liver wet weight and pericardial fat wet weight. **(F, G)** Effect on peroxisome proliferators activated recepotor-γ (or PPAR-γ), **(F, H)** fatty acid binding protein 4 (FABP4), **(F, I)** hormone-sensitive triglyceride lipase (HSL) expression in heart. Vinculin was used as an internal reference protein. **(J)** Correlation between FABP4 and TRPV1 as indicated by quantitative analysis of co-localization immunofluorescence. ^#^ *p* < 0.05 vs. the normal control, ^*^ *p* < 0.05 vs. the model or another treatment group. **(K)** Representative images of FABP4 and CD31 immunofluorescence co-expression in heart (×200 magnification). DAPI stained the nuclei (blue), the red immunofluorescence represents the FABP4 and the green immunofluorescence represents the CD31. ES, electracupuncture stimulation; DAPI, 4′,6-diamidino-2-phenylindole.

Given the potential role of lipids in the progression of ventricular remodeling and myocardial fibrosis, assessing cardiac lipid metabolism levels is instrumental in evaluating cardiac function. The CHF condition was characterized by varying degrees of lipid accumulation in the heart, kidney, and liver, suggesting systemic lipid metabolism abnormalities in the model rats, as illustrated in Figures 2A1–2, 2B1-2, 2C1-2. In reducing steatosis, the digilanid C-ES combination group demonstrated superior efficacy compared to the individual treatments with digilanid C or ES, as indicated in Figures 2A3–5, 2B3–5, 2C3–5. Body weight data may not fully represent the total lipid metabolism anomalies due to the presence of CHF-associated ascites in certain rats, and cardiac wet weight can also be influenced by ischemia and hypoxic changes in the heart. Therefore, liver wet weight and pericardial fat wet weight were additionally observed. The results revealed a linear correlation between liver wet weight and pericardial fat, suggesting that pericardial fat and/or cardiac lipid metabolism levels may more accurately reflect steatosis changes, and that the combination of digilanid C and ES can effectively ameliorate steatosis (shown in Figures 2D–E).

Furthermore, the state of CHF was marked by a significant increase in Fatty-acid-binding protein 4 (FABP4) and an abnormal decrease in hormone-sensitive triglyceride lipase (HSL) and peroxisome proliferators activated recepotor-γ (PPAR-γ), which are cardiac indices related to lipid metabolism, as illustrated in Figures 2F–I. The aberrant expression of these molecular indices was efficiently corrected by the combined treatment of digilanid C and ES. It is proposed that cardiac steatosis is a common feature associated with CHF, and the combined use of digilanid C and ES represents a promising strategy to reduce cardiac lipotoxicity. We also noted that cardiac steatosis was accompanied by a decrease in vascularity, which may contribute to the induction of cardiac steatosis in the CHF state (shown in Figure 2J). The combination of digilanid C and ES has the potential to decrease cardiac oxygen consumption and reduce the effectiveness of free fatty acid generation, as indicated in Figure 2K. This, in turn, could enhance the utilization of lipids as an energy substrate in ischemic tissues and preserve the energy supply capacity of the ischemic myocardium.

### 3.3 ES modulates cardiac energy supply patterns by normalizing lipid accumulation

**FIGURE 3:**
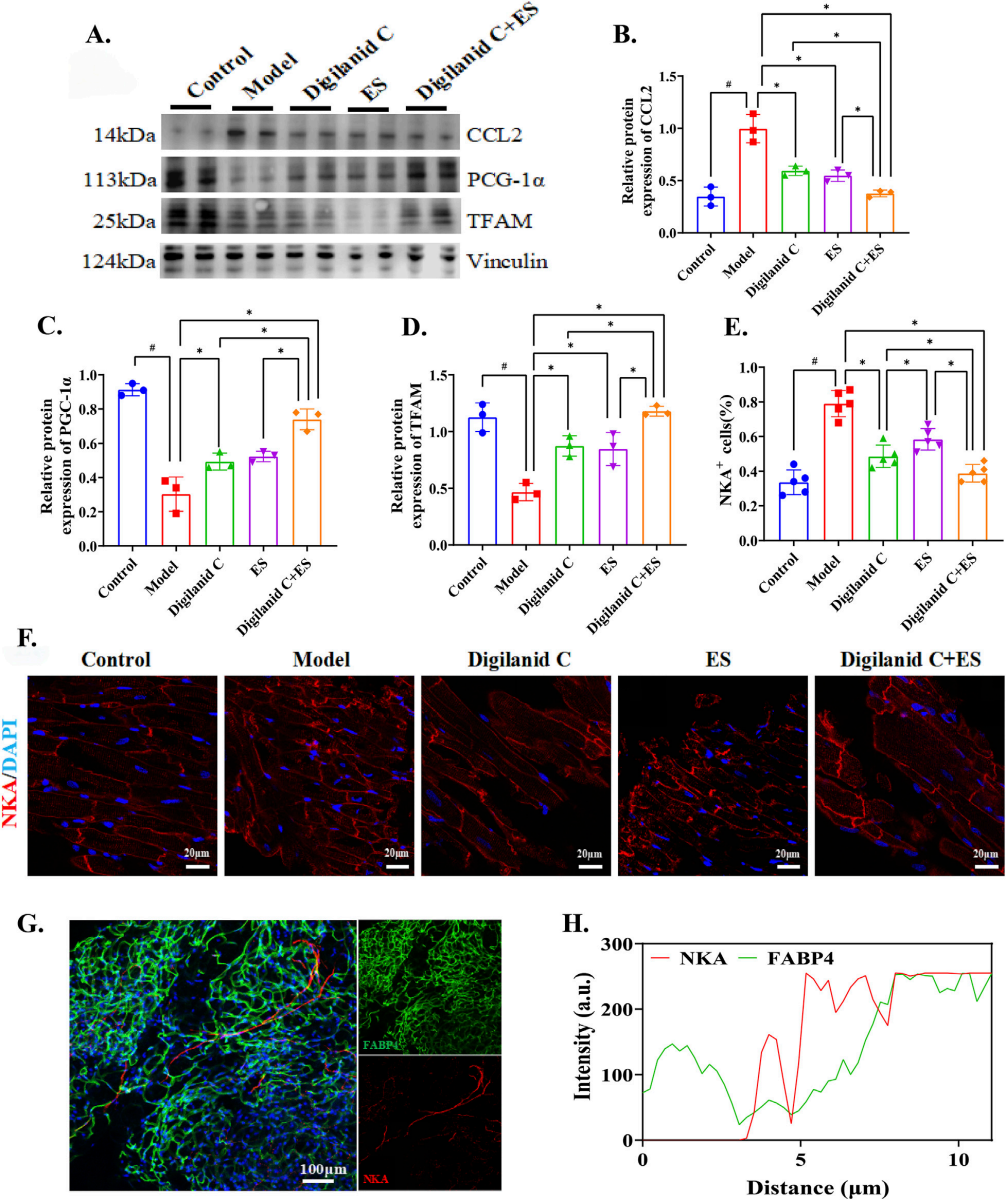
Digilanid C-ES combination to remodeling cardiac energy metabolism. **(A, B)** Effect on chemokine 2 (CCL2), **(A, C)** peroxisome proliferator-activated-recptor-γ coactivator-1α (PCG-1α), **(A, D)** mitochondrial transcription factor A (TFAM) expression in heart. Vinculin was used as an internal reference protein. **(E)** Quantitative fluorescence analysis of NKA-positive cells. **(F)** Representative images of NKA immunofluorescence in pancreas of model group (×200 magnification). DAPI stained the nuclei (blue) and the red immunofluorescence represents the NKA. **(G)** Representative images of FABP4 and NKA immunofluorescence co-expression in heart of model group (×200 magnification). DAPI stained the nuclei (blue), the red immunofluorescence represents the NKA and the green immunofluorescence represents the FABP4. **(H)** Correlation between FABP4 and NKA as indicated by quantitative analysis of co-localization immunofluorescence. ^#^ *p* < 0.05 vs. the normal control, ^*^ *p* < 0.05 vs. the model or another treatment group. ES, electracupuncture stimulation; NKA, sodium-potassium pump; DAPI, 4′,6-diamidino-2-phenylindole; FABP4, fatty acid binding protein 4 3.

An increase in myocardial oxygen consumption suggests that the combination of digilanid C and ES can modulate mitochondrial activity in cardiac tissue, providing cardioprotection through the maintenance of mitochondrial homeostasis. The combined treatment effectively reduced the aggregation of chemokine 2 (CCL2) induced by ischemia or hypoxia, as shown in Figures 3A, B. The levels of mitochondrial transcription factor A (TFAM) and peroxisome proliferator-activated receptor-gamma coactivator (PCG)-1α, two key proteins in controlling mitochondrial activity, also tended towards normalization, as illustrated in Figures 3A, C, D. NKA, abundant in cardiomyocytes, is essential for the heart’s regular pumping activity, and the ability to generate sufficient ATP is critical for optimal myocardial function (Baloglu, 2023). The combination treatment demonstrated superior efficacy in cardiac energy remodeling, with a focused increase in digilanid C’s inhibitory effect on NKA in the myocardium following ES, as depicted in Figures 3E, F. FABP4 can modulate the binding state of free fatty acids when tissues require energy, affecting adipocyte differentiation and body energy metabolism (Prentice et al., 2021). We observed a significant co-expression of FABP4 and NKA, with a positive correlation between their fluorescence intensities, indicating tissue content of the respective proteins. The modulation of abnormal cardiac lipid production by the combination of digilanid C and ES showed a trend consistent with the inhibitory effect of NKA, as shown in Figures 3G, H. Thus, the digilanid C-ES combination could reverse the remodeling impact of aberrant cardiac lipid metabolism on the heart’s energy supply pattern through NKA action, in addition to providing benefits for improving mitochondrial function.

### 3.4 Digilanid C-ES combination mitigates CGCs-Driven neurotoxicity to enhance lipid metabolism and cardiac function

**FIGURE 4:**
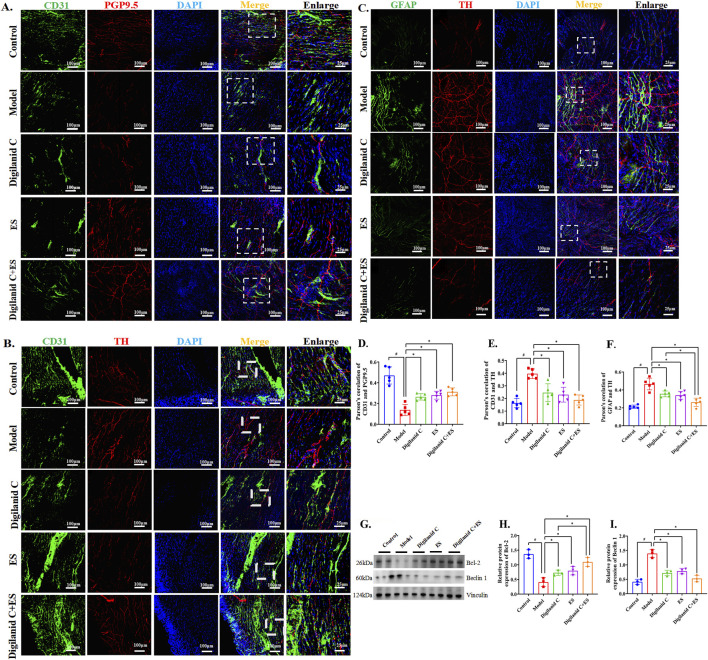
Digilanid C-ES combination drived cardiac glial cells to remodel cardiac neuro-vascular coupling. **(A, D)** Differences in immunofluorescence co-localization of PGP9.5 and CD31 between groups. **(B, E)** Differences in the response of CD31 and TH under different intervention conditions. **(C, F)** Effect on GFAP and TH co-expression after needle-medicine combination intervention. **(G, H)** Effect on bcl-2, **(G, I)** beclin one expression in heart. Vinculin was used as an internal reference protein. ^#^ *p* < 0.05 vs. the normal control, ^*^ *p* < 0.05 vs. the model or another treatment group. DAPI, 4′,6-diamidino-2-phenylindole; ES, electracupuncture stimulation; PGP9.5, proteingene product 9.5; CD31, or platelet endothelial cell adhesion molecule-1; TH, tyrosine hydroxylase; GFAP, glial fibrillary acidic protein.

The findings suggest that myocardial energy supply patterns are influenced by lipid metabolism. To clarify the processes governing lipid metabolism in congestive heart failure, we focused on investigating potential pathways of aberrant lipid accumulation. Lipotoxicity disrupts the functional stability of glial cells, leading to metabolism-associated cell death (lipid death) (Liddelow et al., 2017). At the molecular-morphological level, we found that the cardiac neural network is disrupted in the myocardial ischemia setting of CHF rats, with sympathetic hyperactivity predominating, as shown in Figures 4A–E. Heart lipotoxicity may worsen due to aberrant sympathetic nerve activity. The combination of acupuncture and medication has been shown to be beneficial for restoring the cardiac (sympathetic) neuro-vascular network. Glial cells provide supportive trophic functions for the nervous system. Hyperactivation of CGCs is associated with aberrant cardiac sympathetic nerve activity, suggesting that CGCs can generate neurotoxicity that causes or exacerbates sympathetic hyperexcitability, as illustrated in Figures 4C, F. Compared to digilanid C or ES alone, the combination of digilanid C and ES could partially reverse the pathological symptoms mentioned above and repair the neurotoxicity induced by aberrant autophagy levels, as shown in Figures 4G–I.

### 3.5 Digilanid C-ES combination drives glutamatergic nerve interaction with cardiac fat in a CGCs-Dependent manner

**FIGURE 5:**
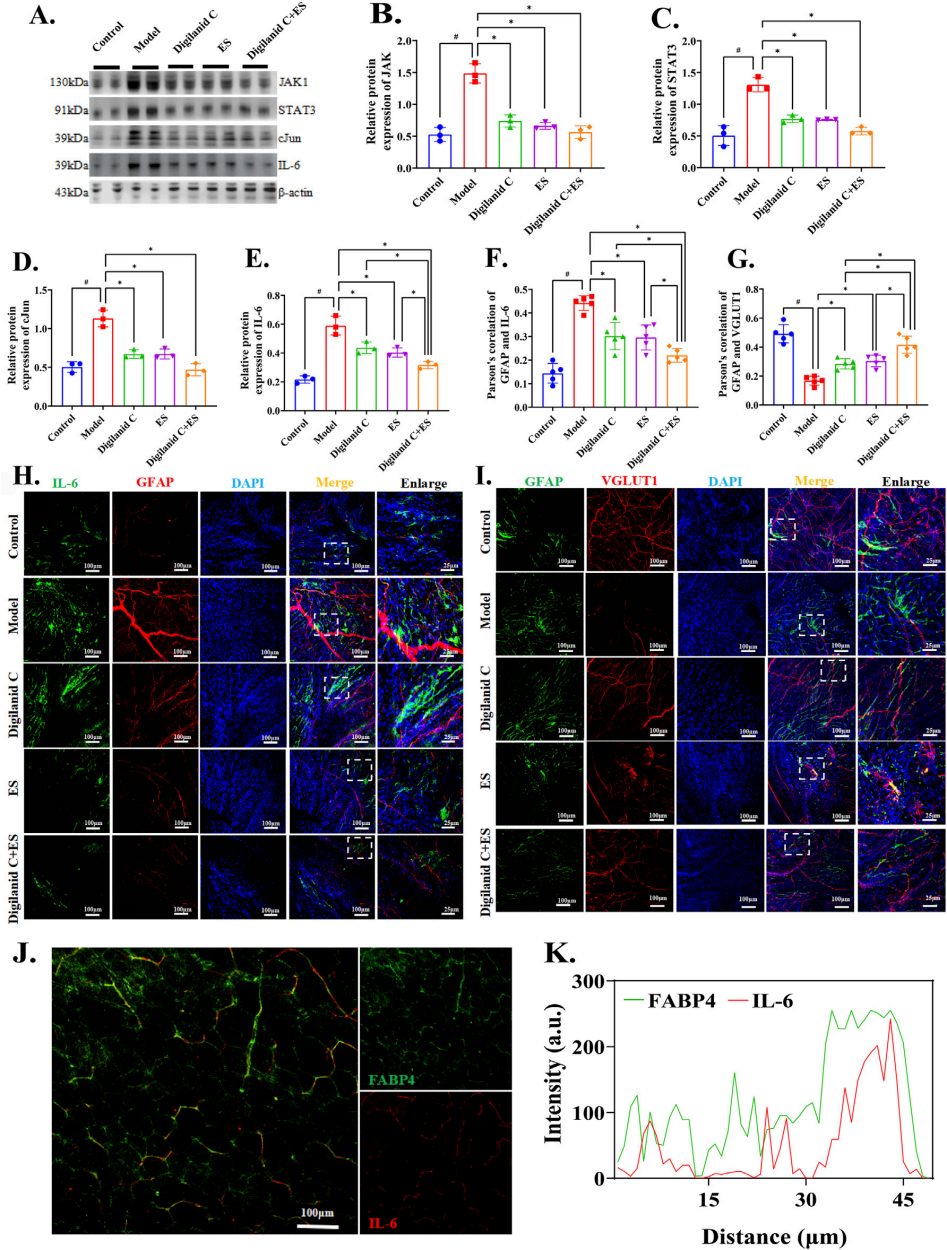
Digilanid C-ES combination harmonizes cardiac glial cell-glutamatergic fibers interactions to ameliorate cardiac lipotoxicity. **(A, B)** Effect on Janus kinase 1 (JAK1), **(A, C)** signal transducer and activator of transcription 3 (STAT3), **(A, D)** cJun and **(A, E)** interleukin 6 (IL-6) expression in heart. Vinculin was used as an internal reference protein. **(F, H)** Differences in immunofluorescence co-localization of IL-6 and GFAP between groups. **(G, I)** Differences in the response of GFAP and VGLUT1 under different intervention conditions. **(J, K)** Effect on GFAP and IL-6 co-expression after needle-medicine combination intervention. ^#^ *p* < 0.05 vs. the normal control, ^*^ *p* < 0.05 vs. the model or another treatment group. DAPI, 4′,6-diamidino-2-phenylindole; ES, electroacupuncture stimulation; PGP9.5, proteingene product 9.5; CD31, or platelet endothelial cell adhesion molecule-1; TH, tyrosine hydroxylase; GFAP, glial fibrillary acidic protein; VGLUT1, vesicular glutamate transporter one.

Further investigation is required to understand how the digilanid C-ES combination targets heart tissues in CHF conditions. We observed the location and regulatory mechanism of CGCs on the injured myocardium. The results demonstrate that CHF is characterized by pathological remodeling of the neurovascular network, an adaptive response triggered by tissue hypoxia and dependent on glial cells. Upregulation of the JAK1/STAT3 signaling pathway is also involved in cardiac fibrosis, abnormal fat accumulation, and the abnormal activation of CGCs. The combination of digilanid C and ES may reduce the over-activation of CGCs during the hypoxic and ischemic phases of CHF by decreasing the signaling pathway associated with JAK1 expression in cardiac tissues, as shown in Figures 5A–E. To reduce cardiac lipotoxicity, further investigation is needed on how the state of CGCs coordinates pathological alterations in CHF and interventional stimuli. Changes in CGCs recruitment and activity were positively correlated with IL-6 activity, as shown in Figures 5F, H. These CGCs can then interact with one another through the glutamatergic nervous system, contributing to the degeneration of cardiac lipids, as illustrated in Figures 5G, I. The digilanid C-ES combination corrects the aberrant glutamatergic connections between CGCs and IL-6, which in turn corrects the lipid over-accumulation driven by IL-6, as shown in Figures 5J, K. In essence, the digilanid C and ES combination reduces cardiac lipotoxicity in a CGCs-dependent manner, involving an IL-6-driven glutamatergic nerve negative feedback loop.

## 4 Discussion

In this study, a clinically relevant rat model of CHF was employed to uncover novel mechanistic insights: 1) myocardial lipid overload and cardiomyocyte lipid accumulation are directly associated with liver wet weight and renal steatosis in CHF rats, reflecting altered cardiac function; 2) cardiac energy supply pattern remodeling, inclusive of adaptive mitochondrial function modifications, is tied to lipid metabolism reprogramming under CHF; 3) IL-6-related pathways are upregulated in response to CHF, modulating the interaction of CGCs with glutamate signaling, thereby mediating cardiac lipotoxicity; 4) these phenomena may be ameliorated by digilanid C alone, ES alone, or a combination of both, with the digilanid C-ES combination demonstrating a significant advantage. In conclusion, we elucidated the role of the digilanid C-ES combination in downregulating the IL-6-related signaling pathway, a key driver in the regulation of cardiac lipid accumulation by CGCs.

The maintenance of adipose tissue homeostasis is intricately linked to the microvascular system (Monelli et al., 2022). Our findings indicate that mitochondrial dysfunction mediated by TFAM is associated with ectopic cardiac fat deposition due to ischemia and hypoxia in the heart during congestive CHF, as illustrated in Figure 2. A complex array of molecular processes, including oxidative stress, mitochondrial damage, disrupted energy metabolism, apoptosis, and inflammatory responses, are implicated in the pathogenesis of ischemic CHF. These biological interactions lead to disruptions in the internal and external environments of cardiomyocytes, exacerbating cardiac damage (Hu et al., 2024). Hypoxia and myocardial ischemia reduce energy metabolism, causing dysregulation of glycolipid metabolism, which ultimately results in cell death and impaired heart function (Tian et al., 2023a). The activity of adipose tissue significantly influences the heart’s vascular niche. FABP4, secreted by adipocytes, is an adipokine crucial for fatty acid transport across the vascular wall. Dysregulated FABP4 expression in obesity can exacerbate cardiac dysfunction, impact vascular endothelial growth factor (VEGF) secretion (Jabs et al., 2018), and lead to vascular disorders. Thus, by modulating FABP4 expression, aberrantly produced VEGF can facilitate metabolic crosstalk on lipogenesis in obese conditions. FABP4 also diminishes the sensitivity of endocrine hormones like insulin and controls the initiation and progression of inflammation, in addition to its role as a lipid regulator. Targeting glycolipid metabolism is an effective strategy to counteract myocardial ischemia damage (Han et al., 2022). Energy-storing molecules such as proteins and lipids undergo metabolic processes like the tricarboxylic acid cycle before being stored as ATP. Consequently, ATP serves as the essential energy carrier linking the anabolism of energy-requiring substances and the catabolism of energy-providing foods. NKA is the primary molecular target of digilanid C. The imbalance in cardiac energy metabolism is exacerbated by cardiomyocyte steatosis, and anomalies in ATP content also affect the expression of NKA in the myocardium, as shown in Figure 3. Therefore, digilanid C’s regulation of NKA will encourage the remodeling of the cardiac energy supply pattern and balance the heart’s pumping function requirement. Mitochondria, as one of the main effectors of ES in regulating energy homeostasis, can be involved in multiple pathological aspects by regulating their own biogenesis, autophagy levels, and other pathways (Hu et al., 2023). The combination of digilanid C and ES could reactivate the heart’s natural energy source, produce new, healthy mitochondria, and efficiently stimulate mitochondrial biogenesis, as depicted in Figure 3.

Despite widespread agreement that aberrant lipid metabolism contributes to CHF, the molecular mechanisms behind cardiac lipid excess remain poorly understood. Sympathovagal imbalance is a common feature in disease-stressed cardiomyocytes (Machhada et al., 2020). Chronic dysregulation of lipid production can facilitate disease progression by exacerbating injuries, as these can disrupt the homeostatic sympathovagal nerve environment (Selejan et al., 2022). Our findings suggest that the combination of digilanid C and ES has applications in repairing cardiac nervous system abundance, decreasing aberrant sympathetic nerve activity, and relying on nerve signaling in the vessel to facilitate communication between distal tissues, as shown in Figure 4. Repairing the neuro-vascular connection will assist the digilanid C-ES combination in enhancing a number of cascade events, including ventricular remodeling and inflammation caused by myocardial blood withdrawal in the condition of CHF (Wagner et al., 2023), thereby regulating cardiac performance. Glial cells modify tissue blood flow reconstruction and vascularization techniques, as well as the development of the tissue nervous system and gliogenesis. Electron microscopy revealed characteristic cardiac glial-like cells (Pauziene et al., 2000). Furthermore, research has shown that these glial cells play a critical role in controlling the functional transmission of information between the parasympathetic and sympathetic nervous systems (Kikel-Coury et al., 2021). Due to glial cells’ trophic support of the nervous system, GFAP-mediated vascular localization is frequently accompanied by nervous system heterogeneity, or the reconfiguration of glial cells, the nervous system, and the vascular module (Yao et al., 2023). Our work indicated that aberrant elevations in sympathetic nerve activity can worsen problems in lipid metabolism in the CHF condition, and that glial cells play a role in altering the cardiac neural system in the CHF disease situation. The digilanid C-ES combination could maintain sympathovagal balance, control the hyperactive condition of CGCs, and alleviate aberrant cardiac function and cardiac fat overload brought on by sympathetic hyperactivity.

Overactivation of glial cells due to inflammation leads to excessive pruning of synapses and dysfunctional neuron circuits (Mancusi and Monje, 2023). CGCs overactivation due to inflammation will worsen the consequences of cardiac neurologic damage, including aberrant cardiac glycolipid metabolism and cardiomyocyte death. Excitatory amino acids or membrane depolarization can cause the production of neuronal IL-6, which has neurotrophic qualities (Juttler et al., 2002). CGCs, pacemaker cells, and fibroblasts, among others, are central to supporting glutamate signaling (Kanemaru et al., 2023). The function of cardiac glutamatergic neurons is supported by CGCs, and we have found a scenario in which IL-6 is still co-expressed in modest levels in the normal condition (Figure 5). This implies that IL-6 is not only produced under pathological conditions but may play a key role as a physiological neuromodulator, induced by neuronal activity and modulating the function of innervated target organs. In chronic hypoxic environments such as CHF, IL-6 expression is upregulated to activate cellular metabolic adaptations and balance energy demands. When sustained hypoxia is not alleviated, IL-6 will stimulate glial cell overactivation to mediate neurotoxicity and ischemic injury (Choi et al., 2023; Liu et al., 2021). STAT3 overexpression in cardiovascular pathogenic contexts is further enhanced by IL-6 (Kawagishi et al., 2022). Conversely, JAK1/STAT3 pathway inhibition by IL-6 silencing suppressed inflammatory and autophagic reactions brought on by lipid accumulation (Nakao and Libby, 2023). Inhibition of the JAK1/STAT3 signaling pathway regulates endothelial barrier function and lipid peroxidation levels (Gao et al., 2023), significantly inhibiting lipid accumulation (Song et al., 2023). CGCs could utilize morphogen signaling through the JAKs/STAT3 signaling pathway to drive differentiation and regulate heart rate and rhythm (Smith, 2023). Potential mechanisms of neural activity by CGCs in the heart rely on VGLUT1’s environment, and VGLUT1 regulates obesity susceptibility (Mizera et al., 2022). It is suggested that the inflammation-induced aggregation of CGCs in the vicinity of cardiac VGLUT1 nerves in the early stage of CHF may be a compensatory response to control cardiac lipid metabolism impairment. And the over-recruitment of CGCs in the late stage would exacerbate the cardiac glutamatergic nervous system injury. The JAK1/STAT3 pathway was downregulated, and the over-activating impact of IL-6 on CGCs was lessened by the digilanid C-ES combination. This improved cardiac function and myocardial steatosis by coordinating the reparative actions of CGCs on the cardiac neural system, particularly glutamatergic neurons.

## 5 Study limitations and clinical implications

The manuscript effectively elucidates the synergistic effect of digilanid C and ES in modulating CGC activity, which is instrumental in enhancing the reconfiguration of cardiac lipid metabolism. The study also sheds preliminary light on the IL-6-mediated JAK1/STAT3 signaling pathway, suggesting its pivotal role in influencing CGC function. Despite these insights, the specific impact of targeted JAK1/STAT3 inhibition on CGCs in cardiac lipid metabolism remains to be elucidated. Future research should focus on the precise silencing of this pathway within cardiac tissues to further delineate its role. The collection of clinical samples was also increased to clarify the scope of application of the findings. This study significantly advanced our comprehension of the pivotal role that CGCs play in modulating cardiac lipid metabolism, thereby offering promising therapeutic targets for the management of lipid metabolism-associated cardiovascular diseases, notably diabetic cardiomyopathy. Concurrently, the research elucidated the potential of ES, when synergized with digilanid C, to augment cardiac function. Our results suggested that only one case of premature ventricular contractions occurred in the digialnid C combined with ES group during the observation period, which may be related to the fact that ES increases the concentration of digialnid C in the target organ. It suggested the possibility that ES improves drug effects to reduce dosage in order to improve the safety of the therapy. This enhancement is achieved through the modulation of metabolic regulatory pathways intrinsic to the heart, presenting an innovative therapeutic strategy for the clinical management of patients with CHF.

## Data Availability

The original contributions presented in the study are included in the article/Supplementary Material, further inquiries can be directed to the corresponding authors.
